# Polymelia in a chimeric Simmental calf: nociceptive withdrawal reflex, anaesthetic and analgesic management, anatomic and genetic analysis

**DOI:** 10.1186/s12917-019-1846-4

**Published:** 2019-03-29

**Authors:** Ute Morath-Huss, Cord Drögemüller, Michael Stoffel, Christina Precht, Patrik Zanolari, Claudia Spadavecchia

**Affiliations:** 1 Department of Veterinary Clinical Science, Anaesthesiology and Pain Therapy Division, Vetsuisse Faculty, University of Bern, Länggassstrasse 124, 3012 Bern, Switzerland; 2 Institute of Genetics, Vetsuisse Faculty, University of Bern, 3001 Bern, Switzerland; 3 Division of Veterinary Anatomy, Department of Clinical Research and VPH, Vetsuisse Faculty, University of Bern, POB 3350, 3001 Bern, Switzerland; 4 Clinical Radiology, Department of Clinical Veterinary Medicine, University of Bern, Länggassstrasse124, 3012 Bern, Switzerland; 5 Clinic for Ruminants, Vetsuisse Faculty, University of Bern, Bremgartenstrasse 12, 109a, 3012 Bern, Switzerland

**Keywords:** Calf, Polymelia, Nociceptive withdrawal reflex, Chimerism, Chronic postsurgical pain

## Abstract

**Background:**

Polymelia is a congenital defect characterized by one or more supernumerary legs. The genetics and aetiology of this condition in cattle have not yet been thoroughly investigated even though several case reports do exist. The model of the nociceptive withdrawal reflex (NWR) has been characterized in several species to study spinal nociceptive processing. It is a polysynaptic spinal reflex that can be elicited by noxious electrical stimulation and recorded by electromyography. Thorough nociceptive examination and preventive analgesic management has not yet been an aspect in the perioperative management of polymelia cases.

**Case presentation:**

A 4-month-old female Simmental calf was presented with notomelia. The animal was in good health and showed no neurologic deficiencies. Preoperatively, computed tomography was performed to gain more detailed anatomical information. To evaluate the sensitivity of the accessory limb, NWR testing was performed and revealed a connection of the afferent reflex pathway of the accessory limb to the efferent of the normal limb. The accessory limb was surgically removed under general anaesthesia. Intensive care included multimodal pain therapy adapted to the pain intensity scored during regular pain assessment. A gross anatomical dissection as well as a genetic analysis of the accessory limb were performed postoperatively. The calf was identified as a chimera.

**Conclusion:**

This calf was successfully relieved of its accessory limb. Chimerism has not been described in the congenital defect polymelia. As the accessory limb was pain sensitive and a common nociceptive reflex pathway was identified, thorough perioperative pain management was performed with the intention to prevent chronic neuropathic pain development.

## Background

Polymelia is a congenital defect characterized by one or more supernumerary legs and can be further categorized into notomelia, cephalomelia, thoracomelia and pygomelia, according to the body region the accessory limb is appearing –the back, the head, the thorax or the pelvis, respectively. Hiraga and Dennis [1] described polymelia as a form of conjoined asymmetric monozygotic twins. Other authors stated it as a cause of heterotopy during embryonal development [2, 3]. To the authors knowledge up to the date of the current report, the genetics and aetiology of polymelia in cattle have not yet been thoroughly investigated, although several case reports do exist [4–6]. Causes for this defect are also unclear, but most likely is to the result of teratogenic impacts during early embryogenesis [7].

The model of the nociceptive withdrawal reflex (NWR) has been characterized in several species to study spinal nociceptive processing and its pharmacological modulation [8, 9]. It is a polysynaptic spinal reflex that can be elicited by noxious electrical stimulation and recorded by electromyography. To the authors knowledge it has not yet been described in cattle.

In the following report we present the case of a calf that was adopted by a social organization and therefore declared as a pet. This fact, and good financial support, allowed thorough preoperative assessment and accurate perioperative intensive care and pain therapy. Additionally this case provided deeper insight into polymelia, emphasizing the importance of individual evaluation and showing that case management needs to be adapted to the actual anatomical and physiologic status.

## Case presentation

### History and preoperative examinations

**Fig. 1:**
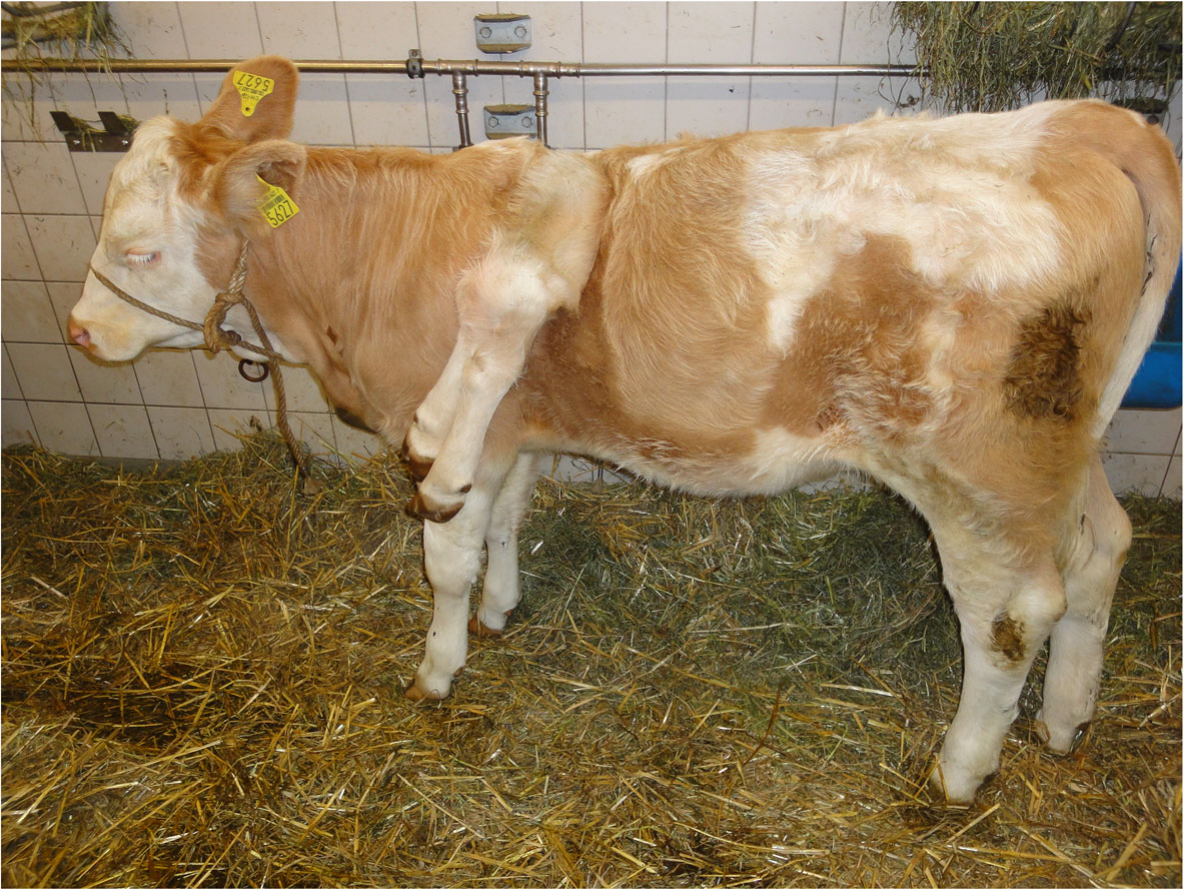
A 4-month-old female Simmental calf was presented with polymelia. The malformation had a common origin attached to the left upper part of the normal scapula and separated into two rudimental lower limbs more distally

A 4-month-old female Simmental calf was presented with polymelia. At first view, the malformation (in the following text termed “accessory limb”) appeared attached to the left upper part of the normal scapula and separated into two rudimental lower limbs distally (Fig. 1). The thoracic part of the spine was scoliotic and lordotic. At presentation the calf was alert and in a good body condition. Except for a slightly increased respiratory frequency (34 breaths per minute) probably due to excitement, the clinical examination revealed an otherwise healthy animal with an adequate state of physiologic development according to its age. Haematology and blood chemistry showed no abnormalities.

As dorso-ventral and latero-lateral radiographs showed significant overlapping of anatomical structures, it was decided to perform computed tomography (CT) to get more detailed information. After premedication with intramuscular (IM) xylazine hydrochloride (0.1 mg/kg; Xylasol 2% Dr. E. Graeub AG, Bern, Switzerland) and intravenous (IV) butorphanol tartrate (0.05 mg/kg; Morphasol 1% Dr. E. Graeub AG, Bern, Switzerland), anaesthesia was induced with IV ketamine hydrochloride (3 mg/kg; Narketan 10% Vetoquinol AG, Bern, Switzerland). and thereafter maintained with isoflurane (Attane™ Isoflurane ad us. vet., Provet AG, Lyssach, Switzerland) delivered in 100% oxygen under pressure-controlled mechanical ventilation.. Monitoring included pulse oximetry, side stream capnography, electrocardiogram (ECG) and oscillometric blood pressure. Computed tomography was performed in sternal recumbency. Anaesthesia was uneventful and recovery was smooth.

### Computed tomography findings

**Fig. 2:**
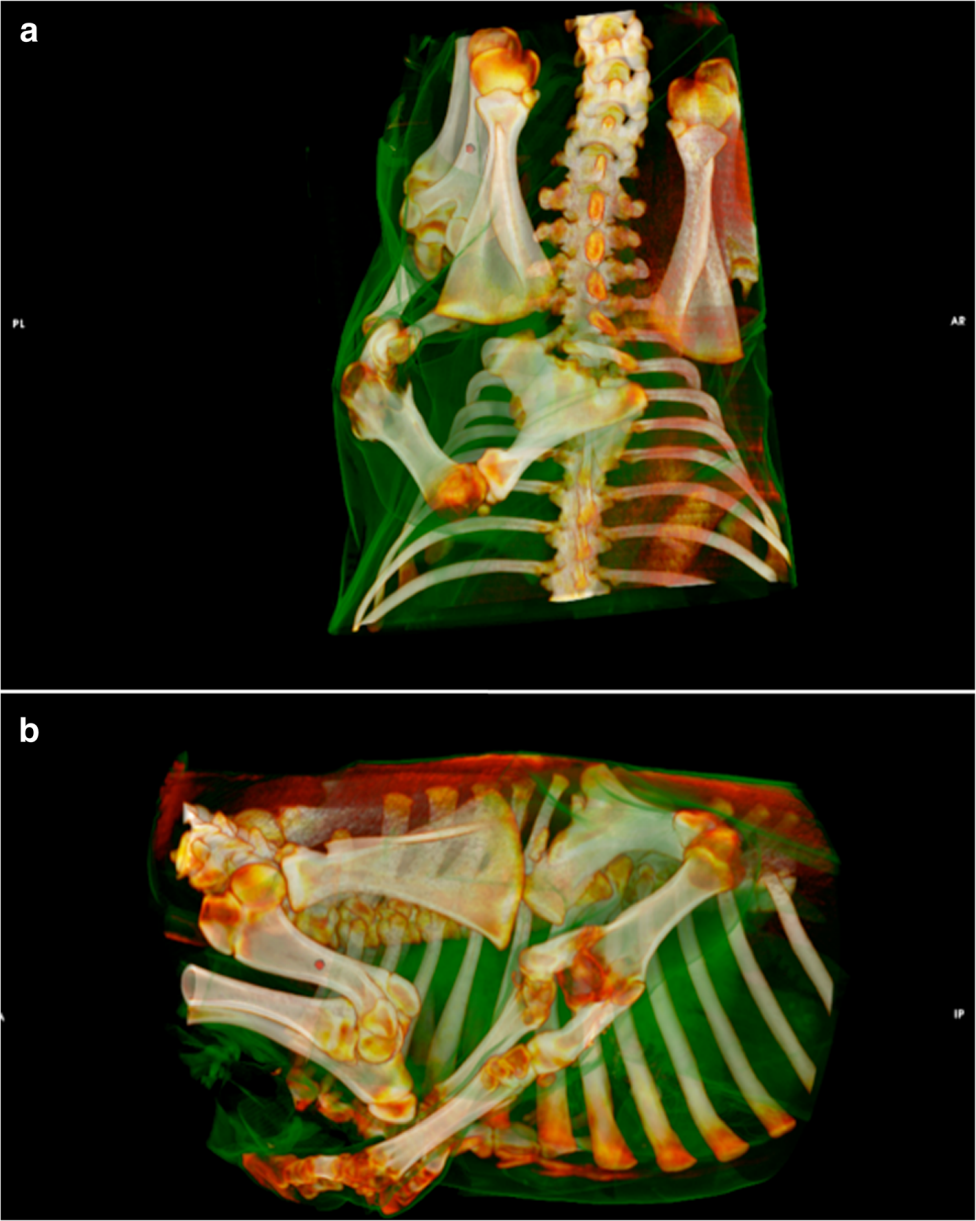
a/b 3D reconstruction of CT images showing the accessory limb and the multiple malformations of the thoracic spine. Dorsal (**a**) and lateral view (**b**) are presented

Computed tomography images (Fig. 2a, b) revealed a polymelia originating from the area of the thoracic spine with formation of a single rudimentary scapula and humerus and paired rudimentary antebrachia and distal limbs. Multiple malformations of the thoracic spine between the 5th and 8th thoracic vertebrae were present. A spina bifida of T6 to T8 with fusion of the right sided spinous processes, a malformation of the dorsal spinous processes of T5 to T8, and a focal scoliosis and lordosis due to an asymmetric T7 hemivertebra were diagnosed. Due to the limited soft tissue contrast of CT, no statement concerning the status of the neuronal and vascular structures could be made.

### Neurologic examination and nociceptive withdrawal reflex testing

A neurologic examination revealed no abnormal findings. As surgical amputation of the accessory limbs was planned, it appeared particularly important to assess whether a noxious stimulus applied to the accessory limb could be perceived and, if this was the case, to describe the nocifensive reaction evoked. Pin prick stimulation as well as pinching did not elicit any withdrawal of the accessory limb, rather stamping of the ipsilateral normal limb. Due to this unexpected reaction it was decided to quantitatively evaluate the nociceptive withdrawal reflex of both the normal and the accessory front limbs to confirm the presence of a common spinal reflex pathway before surgical intervention was started. After shaving, cleaning and degreasing of the skin two self-adhesive stimulation electrodes were placed latero-proximal to the fetlock over the nervus digitalis communis, parallel to the nerve itself, on the normal front extremity and also on the corresponding anatomical location on the accessory limb for sensory nerve stimulation. The anode was placed in the distal position. The paired recording electrodes for surface electromyography were placed over the deltoid muscle of the ipsilateral, normal front limb to record its reflex muscle activity after direct (normal limb) and indirect (accessory limb) electrical stimulation. The ground electrode was placed over the back.

**Fig. 3:**
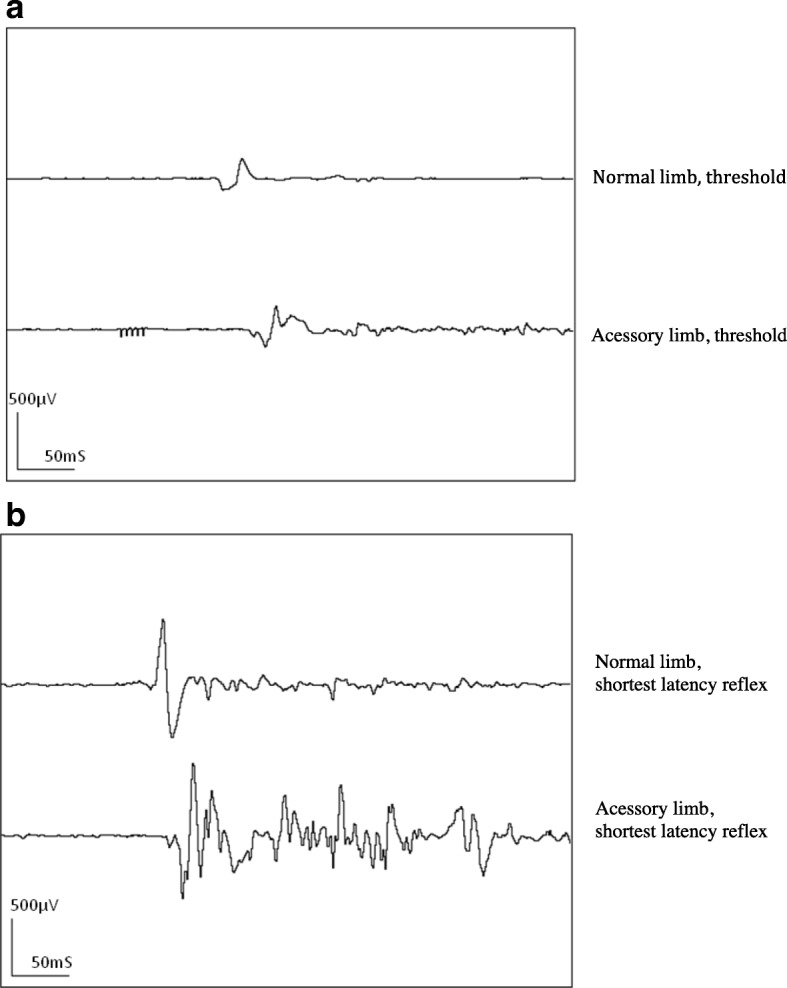
Nociceptive withdrawal reflex recordings from the left deltoid in a calf affected by notomelia. Transcutaneous electrical stimulation of peripheral nerves of both the normal and the accessory limb, at reflex threshold intensity (**a**) (4 mA and 5 mA for the normal and accessory limb respectively) and an intensity eliciting the shortest latency reflex (**b**) (11 mA and 20 mA for the normal and accessory limb respectively)

Normal and accessory limbs were tested following the same order, starting with the normal limb. To define the reflex threshold, a standard stimulus consisting of constant current train-of-five 1 mS pulses at 200 Hz was applied starting at the intensity of 2 mA and increasing in steps of 0.5 – 1 mA until a reflex response was elicited. To confirm the threshold, stimulation was repeated three times at the current where the first electromyographic reflex activity had appeared. Afterwards the current was increased in 0.5–1 mA steps to define the stimulus evoking a reflex with the shortest latency. The results for the normal limb showed initial reflex activity at a current of 4 mA, with a latency of 80 mS, a reflex duration of 40 mS and an amplitude of 81 μV. The shortest latency reflex (31 mS) was seen with a stimulation intensity of 11 mA, a duration of 68 mS and an amplitude of 209 μV. The results from the accessory limb showed an initial reflex at 5 mA, with a latency of 112 mS, a duration of 57 mS and an amplitude of 84 μV. The shortest latency reflex (43 mS) was seen with a stimulation intensity of 20 mA, a duration of 61 mS and an amplitude of 248 μV (Fig. 3).

### Surgical treatment, anaesthetic and analgesic management

Three days before surgical excision of the accessory limb, a therapy with gabapentin (2.5 mg/kg, twice daily, per os; Neurontin 100 mg, Pfizer, Berlin, Germany) was started and continued until discharge from the hospital (24 days of treatment).

Haematology and blood chemistry values were all within physiologic ranges the day before surgery. Food but not water was withheld the evening before surgery, and during the fasting period the calf was kept under maintenance fluid infusion with Ringer lactate solution (2 mL/kg/hr.; Ringer-Lösung “Bichsel”, Grosse Apotheke Dr. G. Bichsel AG, Interlaken, Switzerland). On the day of surgery benzylpenicillin sodium (30,000 IU/kg IV; Penicillin Natrium ad us. vet., Streuli Pharma AG, Uznach, Switzerland), flunixine meglumine (1.1 mg/kg IV; Finadyne ad us vet., MSD Animal Health GmbH, Luzern, Switzerland) and a tetanus antitoxin (3000 IU SC; Tetanus-Serum Intervet ad us. vet., MSD Animal Health GmbH, Luzern, Switzerland) were admistered 30 min before anaesthetic premedication with xylazine hydrochloride (0.15 mg/kg IM) and methadone (0.1 mg/kg IV; Methadon, Streuli Pharma AG, Uznach, Switzerland). Anaesthesia was induced with IV diazepam (0.05 mg/kg; Valium 5 mg, Roche Pharma AG, Reinach, Schweiz) followed by ketamine hydrochloride (3 mg/kg IV). The induction was smooth and endotracheal intubation was performed using a 14 mm endotracheal tube. The animal was placed into right lateral recumbency and connected to a circle breathing system. Volume-controlled mechanical ventilation was started immediately with an inspiratory volume of 10 mL/kg. Isoflurane in 100% oxygen was administered, combined with systemic constant rate infusions of ketamine hydrochloride (0.6 mg/kg/hr) and lidocaine hydrochloride (3 mg/ kg/hr., after a bolus of 1.5 mg/kg over 5 min; Lidocain 2% Streuli ad us. vet., Streuli Pharma AG, Uznach, Switzerland). Methadone boli (0.05 mg/kg IV) were admistered slowly over 5 min every hour and as one IM injection at the end of the procedure. Ringer lactate solution was administered at a rate of 10 mL/kg/hr. Monitoring included ECG, capnography, non-invasive oscillometric as well as continuous invasive (auricular artery) arterial blood pressure measurements. The arterial blood gas analysis results were always within physiologic ranges.

After surgical preparation, a ring block with 20 mL bupivacaine hydrochloride 0.5% (Carbostesin 0.5%, AstraZeneca GmbH, Wedel, Germany) was performed proximal to the planned incision lineTwenty minutes later, an ellipsoid incision was made at the height of the scapulo-humeral joint of the accessory limb, the joint was dissected and the major part of the limb removed. The distal part of the remaining additional scapula, including the acromion, was cut through using an oscillometric saw. The exposed bone marrow of the scapula was covered with a hemostyptic bone wax. The sharp edges of the bone were abraded, and a continuous suction drain was placed. The fascial layer was closed and a wound catheter, previously flushed with bupivacaine hydrochloride 0.5%, was placed subcutaneously with its tip located at the highest point of the wound. Splash local anaesthesia was performed with 20 mL of bupivacaine hydrochloride 0.5%. The skin was closed and the wound covered with a sterile bandage. The calf was then allowed to wake up. Recovery was fast and uneventful. The animal was calm and showed no signs of pain.

Intravenous maintenance fluid therapy was kept up until the next day, when the calf was adequately drinking and eating again. Systemic analgesic therapy included continuation of gabapentin, flunixine meglumine 1.1 mg/kg IV once per day, and buprenorphine hydrochloride (Bupaq ad us. vet., Streuli Pharma AG, Uznach, Switzerland) 0.01 mg/kg IV or IM every 6 h. with a total of 20 mL bupivacaine hydrochloride 0.5% was administered via the wound catheter every 4–6 h for three days. Pain assessment was regularly performed and included assessment of the calf’s overall general condition, occurrence of behavioural changes (respiratory changes, reluctance to move or restlessness, excitement or depression, looking towards the wound, anorexia), recording of physiologic parameters such as heart rate and respiratory rate, reaction to wound palpation (large area/punctual palpation), and subjective pain evaluation (no pain to non-bearable pain). Based on these observations, the interval between the application of local anaesthesia was adapted and additional buprenorphine hydrochloride (0.01 mg/kg IM) was administered on two occasions (during the first morning and the second night post-surgery). After removal of the wound catheter (3 days after surgery) and fading of the last bupivacaine hydrochloride injection, the calf showed increased reaction to wound palpation especially to punctual palpation over a period of 24 h. Nevertheless, it was otherwise in good condition and showed no additional signs of pain when left undisturbed. Flunixine meglumine and buprenorphine hydrochloride therapy was stopped 4 days postoperatively. Mild reaction to wound palpation was present until discharge from the hospital.

Wound healing was adequate, and the suction drain was removed three days after surgery. Antibiotic therapy (benzylpenicillin sodium, 30,000 IU every 8 h IV) was maintained over 5 days and sutures were removed 10 days postoperatively. The calf was discharged from the hospital 21 days after surgery in good health conditions.

### Anatomic dissection

A gross anatomical dissection was performed on the removed accessory limb. The extremity consisted of two left front limbs sharing one common scapula and one common humerus. Further distally, the bones included ossa antebrachii lacking typical characteristics and partially fused ossa carpi. Metacarpal bones and phalanges were typical as well as the metacarpophalangeal and interphalangeal articulations. No discernible muscle tissue could be identified, and tendons were firmly combined with fascia or periost. Only rudimentary tendons of the lateral digital extensor muscle and of the deep digital flexor muscle could be distinguished. On the palmar side, two metacarpal arteries and one single vein were recognizable as well as the equivalents of the common digital dorsal artery, vein and nerve.

### Genetic analysis

**Fig. 4:**
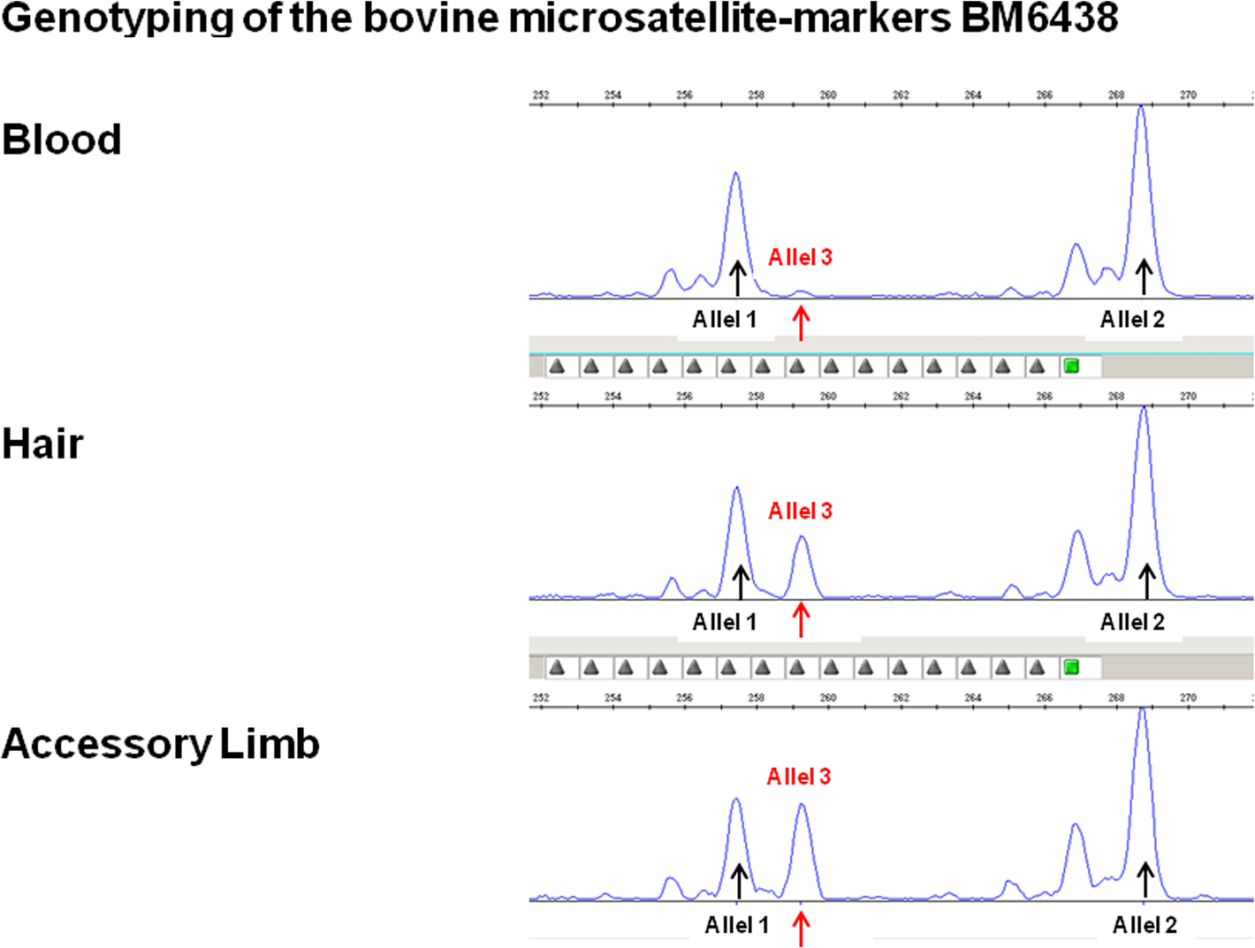
Genotyping of 12 autosomal microsatellite DNA markers revealed at several loci the presence of a third allele. The peak size of this additional marker allele varied between the three DNA sources. The lowest peak size was observed in DNA derived from blood cells, a much higher signal intensity in DNA belonging to hair roots and skin of the additional limb. These findings indicate a chimeric condition

A genetic analysis was performed using genomic desoxyribunucleic acid (DNA) isolated from a skin biopsy of the accessory limb, hair roots from the body, and EDTA stabilized blood of the affected calf. Genotyping of 12 autosomal microsatellite DNA markers using standard methods [10] revealed, at several loci, the presence of a third allele (Fig. 4). The peak size of this additional marker allele varies between the three DNA sources. The lowest peak size was observed in DNA derived from blood cells, and a much higher signal intensity in DNA originating from the hair roots and skin of the accessory limb. These findings revealed the presence of cells with different genotypes, which indicated a chimeric condition of the affected calf known in bovine freemartin pregnancies. A heterosexual twin pregnancy was excluded because a polimerase chain reaction (PCR) showed absence of the *SRY* gene in all available DNA samples.

## Discussion and conclusions

Due to economic reasons, animals with congenital defects that are used for food production are usually euthanized right after birth, or fattened for short periods until slaughtering is possible, or the negative effects of the anatomical changes are no longer tolerable. The calf, presented in this report, was adopted by a social organization and therefore the goal was to treat this animal and improve his life quality on the intermediate-to-long term. Surgical excision of the malformation and a chance to examine this phenomenon more closely from a clinical perspective was possible becauseno neurologic deficiencies and no motoric limitations were present. Additionally, there was unrestricted financial support for essential intensive care.

To the authors knowledge NWR testing in polymelia has not been yet described in veterinary nor human medicine. Our results showed that sensory afferents of the accessory limb were involved in a nociceptive reflex arch with efferents in the normal limb, leading to reflex muscle activity as recorded from the deltoid muscle and visible withdrawal of the normal limb. Possible explanations include a direct autonomous afferent neural pathway connected to the dorsal root of the spinal cord, or an innervation of the accessory limb that is similar to the corresponding dermatome of this region via the lateral ramus of the dorsal ramus of the first thoracal spinal segment. The reflex activity recorded from the deltoid appeared very similar when evoked from either the accessory limb or the normal limb. Reflex thresholds, duration and amplitude of the evoked reflexes were within similar ranges and comparable to those previously obtained in ponies [9]. The latency appeared to be slightly longer in the accessory limb, possibly due to either slower afferent conduction velocity or a longer afferent pathway. Also the reflex recruitment curves appeared to be slightly different, as higher stimulation intensities were necessary to elicit the reflex with the shortest latency, indicating that the accessory limb might have been slightly less sensitive to pain perception than the normal one.

Genetic analysis revealed that the calf was a chimera. Chimera are individuals with two populations of cells, each derived from a different source [11]. This means that the calf presented here can be classified as an asymmetric conjoined dizygotic twin, even though up until now polymelia was defined in cattle as either asymmetric conjoined monozygotic twins or heterotopy. In human medicine, the origin of asymmetric conjoined twins is also considered to be monozygotic either due to incomplete fission of the blastocyte resulting in two centres of axial growth retaining a connection, or fusion of two distinct inner cell masses [12]. Even though the calf presented here had a dizygotic origin, the early fusion of the two cell populations might have led to a neural formation of a common reflex pathway.

The perioperative analgesic management and the risk of possible persistent pain after surgical treatment of polymelia seem, up until now,to be missing in the description of medical care of such cases [4–6, 13, 14]. The fact that the accessory limb was able to perceive pain indicated that there was a risk of neuropathic pain development as seen in cases of routine amputation. Damaged afferent neural structures from the accessory limb could keep on signalling to the spinal cord as if originating from the normal limb due to the fact that the reflex arch was in common and this may lead to chronic pain. Several risk factors for development of chronic postoperative pain were present in this case including: young age, sex, surgical neural structure damage, and risk of postoperative pain [15]. To prevent this pathologic development, multimodal preventive analgesic therapy was initiated, and included systemic as well as local analgesia. Several drugs that have been used for the treatment of this calf are actually not licensed for food producing animals (e.g. gabapentin, methadone, buprenorphine). However, this calf was a special case. It was adopted by an animal sanctuary organisation from Austria and its diagnostics, surgery and hospital stay were documented and filmed as a precedent for animal welfare. When the calf was finally transferred from Switzerland, where it was born and the treatment took place, to Austria, the declaration documents did not declare it as a food animal anymore but as a pet, which was never going to be slaughtered. For all these reasons, we decided to give this animal the best possible treatment we could offer and therefore, we also used drugs that are not licensed for food animals otherwise. The calf was treated with gabapentin prior to surgery. Gabapentin, originally used as an antiepiletic drug, is classified as an anti-hyperalgesic substance. It has been reported as an effective analgesic for neuropathic pain and has been previously described for veterinary use, in small animals, horses and calves [16–20]. Similarly, ketamine, which was used for anaesthesia induction and as an intraoperative analgesic, is classified as an antihyperalgesic drug [21]. It is routinely used in bovine anaesthesia because it is one of the few drugs allowed to be used in food animals by law. Intraoperatively, lidocaine was used as an analgesic adjunct and to reduce isoflurane requirements as reported by Vesal et al. 2011 in a study in calves [22]. For postoperative pain relief buprenorphine was administered, which was previously described by Clutton et al. for perioperative analgesia in lambs undergoing experimental spinal surgery and provided satisfactory intra – and postoperative analgesia [23].

Pain severity has been strongly linked to the development of chronic postsurgical pain and regional anaesthesia is important for postoperative pain reduction [24]. Bupivacaine administered locally provided prolonged desensitization of the damaged tissues, thus preventing local ectopic activity of primary afferents in the immediate post-operative period. In human, pediatric orthopaedic surgery, prolonged bupivacaine instillation via wound catheter in the postoperative period led to lower pain scores and less required additional opioids [25]. Until the day of discharge, the calf showed no signs of pain in the normal limb with exception of the wound area itself which was mildly sensitive to palpation once local anaesthetics were discontinued.

Ten months postoperatively the animal was in good condition without any signs of neuropathic pain. Sensitivity to palpation of the wound area had resolved some weeks after surgery. Due to extraordinary conditions, this calf was successfully relieved from the confinement and health risks of its congenital defect.

Chimerism has not yet known to have been described in polymelia in calves. Even though this genetic constellation has only been documented in this one case, it might give thought-provoking impulses for upcoming cases not only in veterinary medicine. If surgical intervention is planned, a careful preoperative assessment of pain sensitivity in the structures to be amputated and thorough perioperative multimodal analgesia are strongly recommended to reduce the risk of postoperative chronic pain development. Additionally, cases should be evaluated for possible malformations of the vertebral column that could appear in conjunction with polymelia, as it was seen in the presented case.

## Abbreviations

CT: Computed tomography
DNA: Desoxyribunucleic acid
ECG: Electrocardiogram
EDTA: Ethylenediaminetetraacetic acid
IM: Intramuscular
IV: Intravenous
NWR: Nociceptive withdrawal reflex
PCR: Polymerase chain reaction
SC: Subcutaneous
